# Irreversible Electroporation Ablation (IRE) of Unresectable Soft Tissue Tumors: Learning Curve Evaluation in the First 150 Patients Treated

**DOI:** 10.1371/journal.pone.0076260

**Published:** 2013-11-01

**Authors:** Prejesh Philips, David Hays, Robert C. G. Martin

**Affiliations:** 1 University of Louisville, Department of Surgery, Division of Surgical Oncology, Louisville, Kentucky, United States of America; 2 Department of Interventional Radiology Baptist Hospital, Little Rock, Arkansas, United States of America; Northwestern University Feinberg School of Medicine, United States of America

## Abstract

**Background:**

Irreversible electroporation (IRE) is a novel technology that uses peri-target discrete probes to deliver high-voltage localized electric current to induce cell death without thermal-induced coagulative necrosis. “Learnability” and consistently effective results by novice practitioners is essential for determining acceptance of novel techniques. This multi-center prospectively-collected database study evaluates the learning curve of IRE.

**Methods:**

Analysis of 150 consecutive patients over 7 institutions from 9/2010-7/2012 was performed with patients treated divided into 3 groups A (1^st^ 50 patients treated), B (2^nd^ 50) and C (3^rd^ 50 patients treated) chronologically and analyzed for outcomes.

**Results:**

A total of 167 IRE procedures were performed, with a majority being liver(39.5%) and pancreatic(35.5%) lesions. The three groups were similar with respect to co-morbidities and demographics. Group C had larger lesions (3.9vs3cm,p=0.001), more numerous lesions (3.2vs2.2,p=0.07), more vascular invasion(p=0.001), underwent more associated procedures(p=0.001) and had longer operative times(p<0.001). Despite this, they had similar complication and high-grade complication rates(p=0.24). Attributable morbidity rate was 13.3%(total 29.3%) and high-grade complications were seen in 4.19%(total 12.6%). Pancreatic lesions(p=0.001) and laparotomy(p=0.001) were associated with complications.

**Conclusion:**

The review represents that single largest review of IRE soft tissue ablation demonstrating initial patient selection and safety. Over time, complex treatments of larger lesions and lesions with greater vascular involvement were performed without a significant increase in adverse effects or impact on local relapse free survival. This evolution demonstrates the safety profile of IRE and speed of graduation to more complex lesions, which was greater than 5 cases by institution. IRE is a safe and effective alternative to conventional ablation with a demonstrable learning curve of at least 5 cases to become proficient.

## Introduction

 Historically, the most common surgical instrumentation category used for soft tissue removal has been manual surgical instruments (e.g., scalpels, osteotomes, scissors, forceps, etc.). As the technology has evolved, numerous other forms of soft tissue destruction-therapy have been developed, including interstitial ablation. The option to ablate without excisional removal has allowed for expanded treatment options in areas where resection was not feasible. 

 Radiofrequency Ablation (RFA) was developed to augment the surgical excision techniques and is considered one of the treatment options in several disease states, including small hepatocellular cancer and small renal cell carcinomas[1]. The application of RFA has evolved from simple cutting and coagulation of soft tissue (including tumor resection) to the treatment of cardiac arrhythmias[2]. Additional interventions or medical treatment may also be used in conjunction with soft tissue removal to treat the disease, such as oncological drugs for treatment/prevention of tumor growth or recurrence[3]. In addition, it could be used to reduce or eliminate clinical symptoms caused by the disease state and thus improve the quality of life for the patient. 

 Irreversible Electroporation Ablation (IRE) is a novel ablative technology and was provided 510K indications for use for soft tissue ablation by the FDA(first 510(k) in 2006). We have previously demonstrated the safety of IRE around vascular structures in a chronic animal study with good efficacy of IRE in pancreas[4]. Our and others initial Safety Profile analysis has been previously demonstrated the safety profile of the Irreversible Electroporation (IRE) ablation device in liver and pancreas[5-7]. Those initial reports demonstrated initial ablation success and an understanding of ablation recurrences that occurred during the initial cases of each physician and based on size of the lesion to be treated. The optimal learning curve for IRE in soft tissue has not been answered. Various parameters, such as procedure time, blood loss, complication rates and conversion rates, are used to analyze initial experience evolution and to find a cut off point after which the curve plateaus[8]. 

 The aim of this study was to establish the learning curve in the use of IRE for locally advanced unresectable soft tissue. 

## Methods

 Our University of Louisville IRB approved multi-institutional prospectively-collected registry of 150 patients undergoing IRE from 2009 through 2012 was reviewed. Written consent was given by the patients for their information to be stored in the this clinical outcomes database and used for review. There was no standardized protocol dictating patient selection criteria, which were left to the discretion of the treating physician. General exclusion criteria, however, would include general unsuitability to undergo general anesthesia, extensive extrahepatic disease, or multifocal hepatic disease not amenable to complete ablation.

### Ablation Procedure

 IRE was performed using the Angiodynamics Nanoknife system (Angiodynamics, Latham, NY). The Nanoknife system consists of a computer controlled pulse generator that delivers ninety 3000-volt pulses lasting 20-100 microseconds to the peri-tumoral IRE probes[4,9,10]. Treatment planning was based on preoperative imaging with CT scanning in which the tumor dimensions and morphology were measured and input into the pulse generator. The number and spacing of probes needed to create the desired ablation zone was calculated by the generator based on a computer algorithm. Single bipolar, or multiple monopolar probes were used, with typical probe spacing ranging from 1.5 to 2.3cm apart. The probes themselves are 19-gauge diameter and radio-opaque to aid in intra-procedure identification of the probe tip. 

 Access for IRE delivery of the needles was either percutaneously or in the operating room at the time of laparotomy or laparoscopy. Percutaneous cases are performed with CT guidance. As described previously general anesthesia and deep neuromuscular blockade (0 twitches out of a train of 4) is a prerequisite to prevent patient movement when the high voltage pulses are delivered. Careful parallel probe placement, up to 10 degrees parallel) is essential as small deviations can lead to areas of *reversible* electroporation, which are likely to result in tumor recurrence[7]. When multiple probe arrays are utilized, a mechanical guide is employed to maintain proper spacing and alignment. The probes are placed in a manner as to bracket the tumor, rather than violate the tumor itself. The probes must also be completely encased in tissue to prevent arcing.

 Delivery of the pulses is synchronized to the patient’s ECG, which is an incorporated feature of the Nanoknife pulse generator. The pulses are timed to be delivered during the absolute myocardial refractory period 50 milliseconds after the R-wave in order to prevent generation of arrhythmias. Because of this synchronization, the patient must have a pulse rate of under 115. 

Ablation technical success was defined as the ability to successful deliver all planned pulses (at least 90) in accordance with size and dimension of the lesion, as well as on at least 8 week axial scanning to demonstrate a complete ablation without evidence of enhancement. The definition of proximity to major vascular/biliary structures or adjacent organs was defined as <5mm in distance. Adverse events were recorded as per the established Common Terminology Criteria for Adverse Events (CTCAE), version 3.0. All complications were recorded prospectively at all institutions. All attributable complications were defined as all complications that were “related”, “possibly related” and those “without any good causative associations” as decided upon by the treating physician.

### Post-Procedural Follow-up

 Follow-up imaging to confirm ablation success was performed at the time 12 weeks after of IRE therapy and then at three-month intervals. An initial discharge scan was done to evaluate any complications from this new technique and not for treatment efficacy. Ablation recurrence was defined as persistent viable tumor as defined by dynamic imaging in comparison to pre-IRE scan or tissue diagnosis. Ablation success was defined as the ability to deliver the planned therapy in the operative room and at 3 months to have no evidence of residual tumor on cross-sectional imaging of treating-team’s choice such as CT, MRI or PET (if they had a preoperative PET avid scan). Dedicated body-imaging radiologists at each center, who were not blinded to treatment, made radiologic interpretation of recurrence as defined by the RECIST criteria[11]. In cases where imaging was equivocal, biopsies were obtained at the discretion of the treating physician.

### Statistical Analysis

 Patient demographics, tumor characteristics, in hospital outcomes, and local recurrence free survival were examined. Continuous variables were summarized by median and interquartile range (IQR) and compared using the Wilcoxon-Mann-Whitney test while categorical variables were summarized as count (percentage) and analyzed using the chi-squared or Fisher’s exact test, where appropriate. Local recurrence free survival (LRFS) was determined from the time of ablation to radiographic recurrence of the treated lesion. Patients without evidence of recurrence were censored at the time of last follow-up. To determine whether there was an appropriate cutoff in tumor size related to increased risk of LRFS, plots of martingale residuals versus tumor size were examined as described[12]. All statistical analyses were performed using SPSS version 20.0, with p<0.05 considered significant.

## Results

A total of 150 consecutive patients (167 procedures) were enrolled prospectively from 7 centers. The cohort was analyzed for outcomes from the 1^st^ 50 patients treated (Group A), Group B was the 2^nd^ 50 patients treated and Group C the 3^rd^ 50 patients treated.over this time interval and analyzed to see the effect to time in proficiency and also to see progression with respect to more advanced lesions. 

### Comorbidities

The median age for the cohort was 62 (Table 1). Group A (first 50) was a median of 2 years younger than group B (2^nd^ 50) and C (3^rd^ 50,p=0.31). Significant prior cardiac history was in 16 patients, 15 had moderate-severe lung dysfunction, 26 patients had diabetes, 15 had hepatitis, 7 had cirrhosis, 40 were tobacco users and 11 had a history of alcohol abuse. Patients in the latter group (Group C) had lower incidence of cardiac (p=0.03) disease and tobacco use (p=0.006) whereas Group B had more hepatitis (p=0.015). 

**Table 1:** Lesion characteristics among subgroups.

		**Group A (First 50 pts)**	**Group B (Second 50 pts)**	**Group C (Third 50 pts)**	P value*
Comorbidities	PMH Diabetes	7	11	8	0.54
	PMH Cardiac	6	9	**1**	**0.03**
	PMH Pulmonary	3	9	3	0.07
	Tobacco Use	7	**21**	12	**0.006**
	Hepatitis	3	**10**	2	**0.015**
	Prior Abdominal Surgery	28	23	27	0.9
	Chemotherapy	40	**30**	35	**0.04**
	Radiation	16	15	18	0.5
	Intra-arterial therapy	6	12	3	0.08
Liver	Hepatocellular	1	**10**	2	**0.002 (↑B)**
	Met Colorectal	9	9	5	0.4
	Metastatic liver lesions	6	7	4	0.7
	Liver Other	1	1	3	0.3
	Cholangiocarcinoma	1	0	1	0.9
Pancreas	Pancreatic Adenocarcinoma	18	13	18	0.4
	Head	11	8	15	0.21
	Body/Neck	8	5	7	0.6
	Tail	1	0	1	0.9
Kidney	Left	1	0	4	0.6
Lung	Right	4	1	1	0.18
	Left	2	3	1	
Number of lesions(mean)		2.15	2.21	**3.2**	0.07
Vascular invasion(N)		20	18	**31**	**0.00 (↑C)**
Size of lesion	X Axis	2.72	2.55	**3.1**	**0.001 (↑C)**
	Y Axis	2.09	2.16	**3.18**	
	Z axis	1.7	1.65	**2.83**	
	Target Size	3	2.9	**3.9**	**0.001 (↑C)**
Prior Ablation(RFA)		1	4	5	**0.04(↓B)**

* p<0.05 significant

**↑ stands for higher in**

**↓ stands for lower in**

### Tumor characteristics

167 procedures (150 patients) included 66 liver (39.5%), 6 kidney (3.6%), 59 pancreas (35.5%), 18 lung (10.8%) and 18 other lesion ablations (10.8%) including kidney, prostate, esophagus, sacrum, mediastinum and adrenal (Table 1). All 3 groups were evenly matched on metastatic colorectal cancer (p=0.4), benign lesions, cholangiocarcinoma (p=0.6), other metastatic lesions (p=0.65) but Group B had more HCC (p=0.002) while A and C had more pancreatic lesions (p=0.04). AFP levels in patients who expressed them were higher in Group A (p=0.00). Location of the lesions in the liver were relatively evenly distributed. Pancreatic lesions were mainly adenocarcinomas 52(31.2%) and a few periampullary lesions 7(4.6%). Lung lesions were relatively evenly distributed and included SCC lung primary (p=0.13), adenocarcinoma (p=0.6) and metastatic lung disease. 

### Lesion characteristics and treatment

Numerically the number of patients with discrete lesions in Group A, B and C were 46, 42 and 43 while 4, 8 and 7 patients respectively had lesions that were too numerous to characterize (p=0.21). The mean number of lesions (C=3.2 vs A=2.15 and B=2.21, p=0.24) and average number of treatments was higher in Group C (C=1.31 vs A=1.22 and B=1.1, p=0.4) but this did not reach statistical significance. The dimensions of the lesions in the X, Y and Z axes were significantly higher in Group C (p=0.001). The median total target size (3.8cm for the whole cohort) as well as lesions > 3 cm were also statistically significantly higher in the latter Group C (p=0.009/0.016). Vascular invasion was noted in 77(51.3%) on radiological review and confirmed during procedure, with preponderance for Group C (n=31 versus 20/18, p=0.00). Peritoneal disease (5), advanced cirrhosis (5), nodal disease (8) and lesions amidst abnormal parenchyma were equally distributed

### Adjunctive therapy

Patients in Group B had a significantly lower incidence of prior chemotherapy (p=0.04). All three had similar incidence of radiation therapy (p=0.5), intra-arterial therapy (p=0.08) and prior hepatic resections (0.7). Group A had a lower incidence of prior failed RFA ablations (p value-0.05). 

### Procedure

Median procedure time was 152.5 minutes and IRE delivery time was 28 minutes per target and both were significantly higher in Group C (239 minutes and 34.6 minutes, p=0.001) (Table 2). The procedure time was significantly higher in patients undergoing additional procedures, usually while undergoing laparotomy (p=0.03). The median number of probes used per ablation was 3(mean 3.35), which was also significantly higher in Group C (3.48, p=0.03). Group B had a higher number of percutaneous ablations and less open (laparotomy) IREs compared to Group A or C (p=0.001). The total time for needle placement was significantly decreased from Group A (mean 40 min), to Group B (mean 25min) , and Group C (mean 20min) (p=0.01). The key break point for a significantly decreased in needle placement time at each institution was 7 patients (OR 2.9, p-0.01). Associated complex hepato-biliary and pancreatic procedures as well as palliative procedures were significantly higher in Group C (p=0.02). 

**Table 2:** Procedure parameters between groups.

	**Group A**	**Group B**	**Group C**	P value*
**Percutaneous**	25	**47**	29	**0.001**(↑B)
**Open- Laparotomy**	27	**11**	22	**0.001**(↓B)
**Major hepato-pancreatic-biliary surgery**	11	4	**16**	**0.02**(↑C)
**Other associated procedures**	29	**15**	30	**0.00**(↓B)
**Pancreatic ablation**	21	**14**	24	**0.041**(↓B)
**Liver lesion ablation**	19	**31**	16	**0.048**(↑B)
**Target Size**	3.02	2.92	**3.98**	**0.003**(↑C)
**Target >3cm**	17	24	**31**	**0.004**(↑C)
**Number of probes**	3.01	3.45	**3.48**	**0.03**(↑C)
**Procedure time**	130	169	**239**	**0.00**(↑C)
**Needle placement time**	25.8	17.9	**48.6**	**0.01**(↑C)
**IRE delivery time**	15.7	32.4	**34.6**	**0.00**(↑C)
**Hospital stay**	5.2	2.6	4.8	0.09
**Incomplete Ablation**	3	4	5	0.2
**Complications**	14	11	17	0.24
**High Grade complications**	6	3	7	0.3
**Attributable complications**	9	5	8	**0.02**(↓B)
**Peri-operative deaths**	2	0	0	**0.01**(↓B,C)
**Recurrence of disease**	11	11	12	0.7
**Local Recurrence**	5	4	4	0.9

* p<0.05 significant

**↑ stands for higher in**

**↓ stands for lower in**

### Complications

A total of 2(one related in Group C) peri-operative deaths(mortality rate 0.6%) were seen in this study (Table 3). Analysis was performed of the related death and a diagnosis of pancreatic cancer and size of the lesion was noted to be a significant association whereas vascular invasion, size of the lesion and prior chemotherapy were not noted to be statistically significant factors. 

**Table 3:** Factors affecting complications.

	**Complications**	**Attributable complication**	**High-Grade Complication**	**P value#**
**Liver(77)**	7(8.5%)	5(6.1%)	2(2.4%)	**0.00/ 0.01/0.00** *
**Pancreas(91)**	39(41.5%)	17(18.1%)	17(18.1%)	0.00/0.07/0.001
**Open(100)**	44(44%)	21(21%)	21(21%)	**0.00/ 0.00/ 0.00**
**Percutaneous(141)**	9(6.3%)	5(3.5%)	2(1.4%)	**0.00/0.00/0.00** *
**Major abdominal surgery(73)**	29(39.7%)	10(13.7%)	13(17.8%)	**0.00/0.01/0.007**
**Prior other ablation(21)**	0	0	0	**0.1/0.1/0.1** *
**Radiation(68)**	27(40%)	11(15.4%)	12(17.6%)	0.00/0.04/0.01
**Intra-art Rx(28)**	3(10.7%)	3(10.7%)	2(6.8%)	0.8/0.7/0.7
**Chemo(106)**	30		11	0.15/ 0.4
**HCC(26)**	1(3.8%)	1(3.8%)	0	0.02/0.2/0.13*
**MCRC(33)**	3(9.1%)	3(9.1%)	0	0.05/0.05/0.06*
**Pancreatic adenocarcinoma(81)**	39(46%)	16(18.8%)	16(18.8%)	**0.00/0.00/0.00**
**Vascular invasion(105)**	43(36%)	18(15.4%)	21(17%)	**0.00/0.03/0.00**
**PMH cardiac(17)**	5(23.8%)	1(4.8%)	1(4.8%)	0.7/0.1/0.4
**PMH Diabetes(40)**	15(37.5%)	7(17.5%)	8(20%)	0.007/0.1/0.01
**Tobacco use(54)**	18(28.1%)	8(12.5%)	7(10.9%)	0.1/0.5/0.5
**Length of stay**	10.5	10.1	14.8	**0.00/0.00/0.00**
**Size of lesion(X, Y Z Axes, cm)**	3.4x3x2.6 vs. 2.6x2.4x2.2	3.4x3x2.4 vs. 2.7x2.5x2.2	3.4x3x2.7 vs. 2.7x2.5x2.2	**0.00/0.3/0.01**

^#^ p<0.05 significant

* Lower Complication rate

There were a total of 84 complications in 42 patients(14, 11 and 17 patients in group A, B and C; p=0.2) with a morbidity rate of 29.3%. This similarity of complications is despite Group C having larger lesions treated, as well as great percentage of associated palliative procedures performed. The median complication grade was 2. The most common complications were peri-procedure nausea/ vomiting(6), infection(6), and severe pain(5). There were no significant cases of IRE-induced cardiac dysrhythmias, but one case of intraoperative asymptomatic self-limiting ST segment depression was seen in Group C. The rate of high-grade complications(> grade 3) in this study was 10.6%(16 patients: 6,3 and 9 each in Group A, B and C; p=0.3). Among the high grade adverse events were 3 cases of DVT/PE, one case of bile leak and 2 of biliary strictures, 2 cases of bleeding requiring transfusions and 1 portal vein thrombus. Since there were a significant number of patients who underwent associated procedures, analysis of complications attributable to the procedure was also done. 20 patients(13.3%) had complications attributable specifically to IRE, which was lower in Group B(p=0.02). The attributable high-grade complication rate was 4%. 

Medical co-morbidities including did not statistically affect the complication rates or high grade complications. Pancreatic lesions(p=0.001), open surgery (other associated procedures, p=0.001) and prior history of radiation(p=.001) were predictive of complication. Procedure times, high current situations and number of lesions for IRE did not significantly impact complication rates(p=0.4) but a longer length of stay (6 days more) was noted in patients with complications. 

Interestingly on comparison of complication rates between the three groups with respect to type of access showed that open procedures were associated with a significantly higher complication rate in Group B compared to Group C(80% versus 63%, p=0.001). Similarly complications were higher in Group A and B compared to Group C when looking at pancreatic IRE(p=0.02), with all of these patients treated through an open incision. In Group A liver lesions, the complication rates were 4/19(21%) whereas in Group B and C they were significantly lower at 2/47(4.2%, p=0.03). The median hospital stay for this cohort was 1 day (Mean=4.2 days). Group B had shorter hospital stay compared to Group A or C (p=0.09 NS).

### Incomplete Ablation

A total of 12 patients were felt to have been incompletely ablated. These were 3, 4 and 5 cases in groups A, B and C (p= 0.2). These included 2 pancreatic lesions, 2 lung, once presacral and 2 had to be aborted due to and technical difficulties. 

### Recurrence

In our median follow-up period of 18 months, 35(31%) patients had recurrence, which was evenly distributed between groups (p=0.7) Mean time to recurrence was 6.7 months in those patients that had recurrence (local and remote). Local recurrence was seen in 13(10.7%) with a mean recurrence time of 6.7 months and local recurrence was also similar among the subgroups (p=0.9). The recurrences were diagnosed with CT scan (n=23), MRI (n=5) and PET CT (n=7, for patients who had a PET positive lesion pre-procedure). Three patients had biopsy confirmation. Six patients had evidence of recurrence at their 3 month follow-up imaging and were called persistent disease and underwent re-ablation. Analysis showed that presence of complications (p=0.001), high-grade complications (p=0.04) and incomplete first treatment (0.03) increased tumor recurrence. Tumor size not impact local recurrence (p=0.1) but increased recurrence of disease at site distant to primary ablation site (p=0.02). Also there was an increase in recurrence rates of tumors with vascular invasion (16.9% vs 4.8%, p=0.06) but this did not reach statistical significance. There was also and decrease in the number of recurrences per treating physician in relation to the number of cases performed. Ablation recurrences for all physicians in their first 10 cases was 26%, with a significant drop in ablation recurrences to 6% for cases 11 or greater (p=0.01).

## Discussion

Irreversible electroporation is a relatively new and evolving technique in soft tissue tumor ablations[13]. Its advantages compared to RFA and conventional ablation techniques is its non-thermal delivery mechanism and was first developed in conjugation with chemotherapy. When properly applied, theoretically, it only affects the target tissue. Proteins, the extracellular matrix, and critical structures such as blood vessels and nerves are all unaffected and left healthy by this treatment[14]. This expands the scope of treatment of lesions near major vascular/ biliary/ urinary structures better than conventional thermal ablative techniques. The major disadvantage is the need for general anesthesia (deep paralysis) for its conduct[5].

This is the largest study in published literature on this new technique. Also it is a unique study in its varied organ sites and access techniques for ablation. The cases were divided into 3 groups (group A, B and C) over time to facilitate analysis and to tease out a possible learning curve inflection point. This was a similar strategy as employed by other authors[14-18] but with 3 subgroups[19]. The 3 groups were comparable with respect to age and overall co-morbidities but Group A and B had fewer pancreatic lesions, fewer lesions with vascular invasion and more liver lesions.

The study group was a median of 62 years old, which is comparable to other tissue ablation experiences [17,18]. Cardiopulmonary disease (20.7%)and a history of tobacco use (27%) was higher than with other reports of early RFA studies[6] but the incidence of cirrhosis was lower than previously reported which is a reflection of the distribution of lesions among other organs[17].. Majority of the cases involved liver and pancreatic lesions (75). 61% of the procedures were performed percutaneously and 36% open. As far as the distribution of liver lesions there were more metastases ablated than primary liver tumors, which is similar to recent western literature for RFAs [20]. A significant number or patients (22%) had other concurrent major abdominal surgery including palliative procedures as well as resections. This is in contradistinction to the early learning curve analyses of RFA, which evaluated primarily percutaneous RFAs without any other associated procedures. We chose to present our consolidated data to reflect the individual preferences of the operators as well as to evaluate this new procedure in its varied access and organ-specific approaches. The earlier groups had a significantly higher number of liver lesions and percutaneous ablations, while Group C had significantly more open procedures; more associated major procedures and significantly more pancreatic lesions. 

A significantly higher number of tumors were noted to have a vascular invasion, which was statistically more prevalent in Group C. This was much higher than most studies of similar ablative techniques [17,18,20] and is reflective of more advanced disease and the advantage of IRE’s non-thermal action. Lesions with significant vascular involvement or involvement of biliary, collecting system, bronchial tree and neural structures have long been noted to be significant contraindication for traditional thermal induced ablation techniques. IRE offers a suitable alternative and in this study we found a large number such anatomically hostile lesions. In spite of there was only one vascular complication of a portal vein thrombosis worsening in a patient with preexisting portal vein. The vascular complication rate was 1.3% (1/77 cases) in patients with vascular invasion and 0.6% in this cohort, which is lower than precedents[17,18,20].

The mean number of lesions and the size of the lesions with respect to X, Y and Z axes as well as the median target size was significantly higher in Group C. The overall mean number of lesions was also similar to comparative studies. Number of probes used for IRE, which is a surrogate for complexity and size of the lesions, was also higher in Group C.

 The procedure time at 152 minutes was significantly longer than most in relatable thermo-ablative studies and similar to IRE studies, even after controlling for associated procedures[6]. Delivery of 90 pulsed treatments with an average of 2 mins per treatment lends to a significantly longer treatment time than RFA and microwave, and is once of the disadvantages of IRE. The operative time was significantly higher in the last group in part due to addition of complex procedures and larger lesions. 

Incomplete ablation was noted in 12(4.7%, similar across groups) of lesions that were either incompletely ablated or found unsuitable. As noted in our previous experience, pre-operative dynamic imaging which is used to plan these treatments, sometimes underestimates the degree of involvement with surrounding structures or the size, especially for pancreatic and retroperitoneal structures. A majority of our patients had a post-procedure imaging and one at 3 months to evaluate the response to treatment and 10.1% of patients had evidence of persistent tumor on repeat imaging. 11 of whom underwent re-ablation successfully. This is a rate that is similar to initial RFA learning curve experiences[20] but most studies did not report this rate. Most patients had a good level of paralysis and there were no significant anesthesia complications intraoperatively, compared to similar studies with IRE[6] and RFA[20]. 

The complication rate in this cohort was 29.3%, which is significantly higher than similar studies (reported complication rates of (6-16%) in RFA and microwave. Complications were also graded as related to the procedure, unrelated, possibly related and related to concurrent procedures. For purposes of analysis attributable complications were all complications that were “related”, “possibly related” and those “without any good causative associations”. The attributable complications rate was 13.3%, which is more congruent with similar precedents. On comparing only percutaneous IRE and their complication rates, the complication rate was comparable at 6.8%[17]. High-grade complications were noted in 16(10.6%) with 6(4%) attributable complications attributable to IRE. No specific gradation of complications and auditing of re-interventions were seen in similar studies, such that a comparison could not be made. There were no cases of cardiac arrhythmias in this study, which is lower than previous literature[6].

Risk factors associated with an increase in complication rates were pancreatic lesions, HCC (compared to MCRC), open surgery with associated procedures and a prior history of radiation. In the evaluation across all three groups (A,B, anc C) analysis, over time larger lesions, more complex tumors and tumors with significantly vascular invasion were ablated the complication rate and high-grade complication rates between the 3 groups were statistically similar. 

Two (1.3%, one related 0.6%) peri-operative deaths are similar to reported studies of similar ablation procedures[17]. One was unrelated, LGI bleed (documented from another source). The other was a patient with advanced 5.4x 4.3x 4cm pancreatic body adenocarcinoma with progression in chemotherapy. An open bypass and palliative IRE was planned. Post-procedure worsening renal failure and progression of pre-existing portal vein thrombus was noted. Pancreatic cancer diagnosis and large size of the lesion were noted to be significant associated factors. 

Local recurrence was seen in 10.7% of patients ablated and is comparable to similar studies. These lesions were larger lesions with greater number of anatomically hostile features including vascular invasion and we think that this is an acceptable recurrence rate given the follow-up period of upto 3 years. Incomplete first treatment (even if subsequently addressed), adverse events at ablation, open surgery (at either ablation site or surgical extirpation site) were associated with increased recurrence. There was a trend to increased recurrences with larger lesions, lesions vascular invasion and pancreatic lesions but this did not reach statistical significance. There was no significant difference in recurrence rates between the three groups.

Overall in this study, there was a significant maturation of the procedure over time with larger tumors, lesions with greater organ involvement and vascular invasion and a greater number of concurrent major procedures were performed especially in the last 50 cases. Independently these were all significant risk factors in complications rates as well as local recurrence but among groups, the complication rates plateaued after the first 100 cases. Recurrence rates and complete ablation rates, surrogates for success of local therapy were similar. Collectively, after the first 100 cases there was significant advancement to more complex procedures with comparable outcomes and is our inflection point on the learning curve. This temporal advancement, albeit a sign of greater confidence in the procedure, must be tempered by the fact that in our analysis a cut-off target size greater than 3 cm increased the risks and procedure times. 

**Figure 1:**
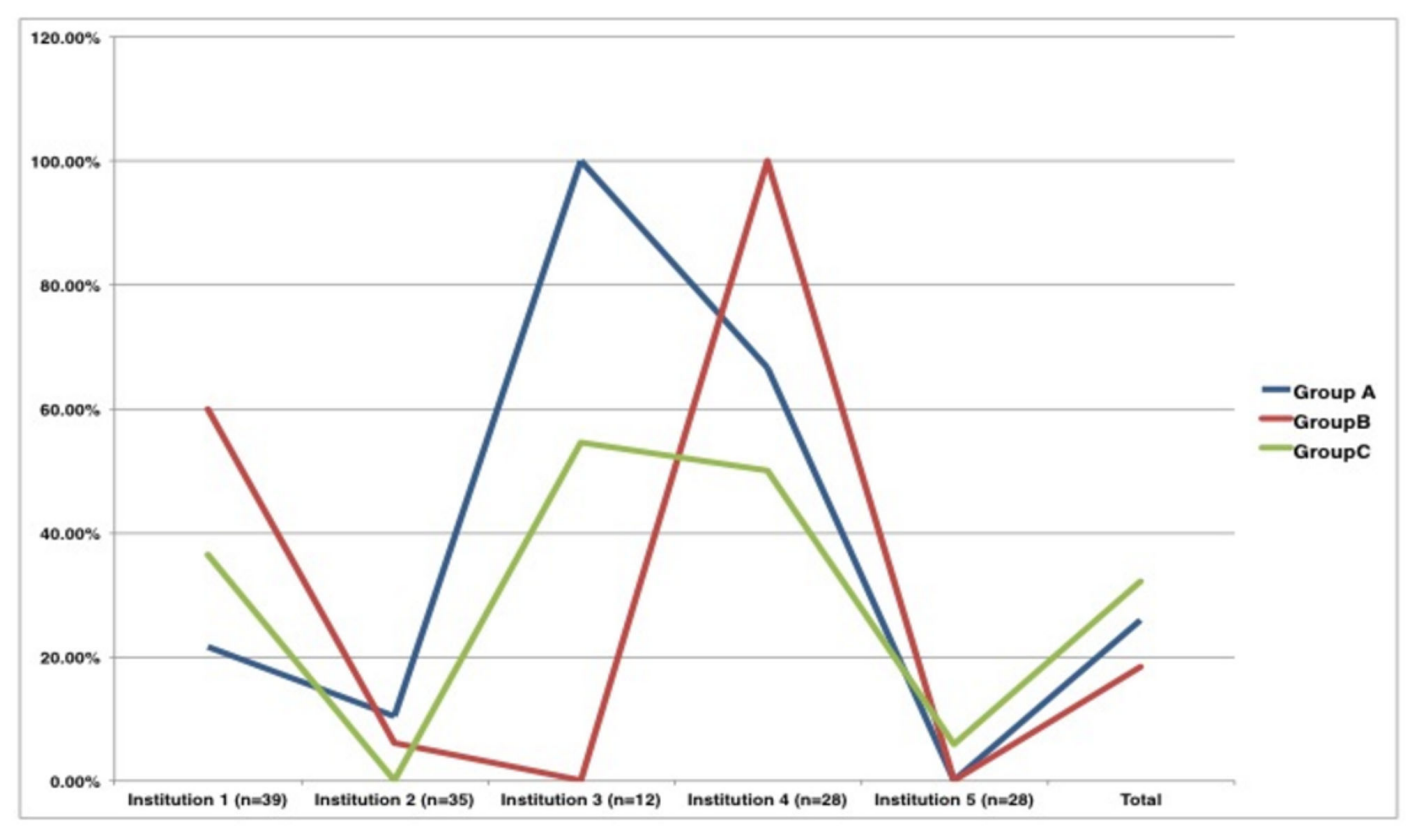
Complication rates (in %of total) among institutions over time. X-Axis: Institutions. Y-axis: Complication-rate in percentage. Lines: Blue- Group A (1^st^ 50); Red- Group B (2^nd^ 50); Green-Group C (last 50).

With regards to individual maturation in the procedure, we also divided the procedures between various institutions to look for individual maturation over time and as Figure 1 shows the majority complications plateaued or dropped after Group B, which is at a median of 5 cases. 

### Summary

IRE is a new non-thermal based electroporation technique of tissue ablation, which acts by changing the membrane properties allowing cell death. Accurate mapping and image based guidance can led to precisely targeted tissue destruction. Since it is not thermal based it avoids the “sump” limitations and can be used for lesions abutting thermo-sensitive or thermal-limiting structures such as vascular, biliary, urinary and nervous structures. Our study demonstrates that IRE could be successfully performed in a majority of the cases without major adverse events. Institutional and individual preferences colored the mode of access and other associated procedures that were performed simultaneously. With time, more complex treatments of larger lesions and lesions with greater vascular involvement was performed without a significant increase in adverse effects or impact on local recurrence. The evolution of this procedure over time in this initial experience demonstrates the safety profile of IRE and the relative speed of graduation to more complex lesions in a relatively short span of time, which in our analysis collectively was 100 cases, and by institution 5. IRE is a safe and effective alternative to conventional ablation with a demonstrable learning curve.
